# Evaluation of serial crystallographic structure determination within megahertz pulse trains

**DOI:** 10.1063/1.5124387

**Published:** 2019-12-04

**Authors:** Oleksandr Yefanov, Dominik Oberthür, Richard Bean, Max O. Wiedorn, Juraj Knoska, Gisel Pena, Salah Awel, Lars Gumprecht, Martin Domaracky, Iosifina Sarrou, P. Lourdu Xavier, Markus Metz, Saša Bajt, Valerio Mariani, Yaroslav Gevorkov, Thomas A. White, Aleksandra Tolstikova, Pablo Villanueva-Perez, Carolin Seuring, Steve Aplin, Armando D. Estillore, Jochen Küpper, Alexander Klyuev, Manuela Kuhn, Torsten Laurus, Heinz Graafsma, Diana C. F. Monteiro, Martin Trebbin, Filipe R. N. C. Maia, Francisco Cruz-Mazo, Alfonso M. Gañán-Calvo, Michael Heymann, Connie Darmanin, Brian Abbey, Marius Schmidt, Petra Fromme, Klaus Giewekemeyer, Marcin Sikorski, Rita Graceffa, Patrik Vagovic, Thomas Kluyver, Martin Bergemann, Hans Fangohr, Jolanta Sztuk-Dambietz, Steffen Hauf, Natascha Raab, Valerii Bondar, Adrian P. Mancuso, Henry Chapman, Anton Barty

**Affiliations:** 1 Center for Free-Electron Laser Science, Deutsches Elektronen Synchrotron, Notkestrasse 85, 22607 Hamburg, Germany; 2 European XFEL, Holzkoppel 4, 22869 Schenefeld, Germany; 3 Center for Ultrafast Imaging, Universität Hamburg, Luruper Chaussee 149, 22761 Hamburg, Germany; 4 Deutsches Elektronen Synchrotron DESY, Notkestrasse 85, 22607 Hamburg, Germany; 5 Institute of Vision Systems, Hamburg University of Technology, Vision Systems E-2, Harburger Schloßstr. 20, 21079 Hamburg, Germany; 6 Department of Physics, Universität Hamburg, Luruper Chaussee 149, 22761 Hamburg, Germany; 7 Department of Chemistry, University at Buffalo, 359 Natural Sciences Complex, Buffalo, New York 14260, USA; 8 Hauptman-Woodward Medical Research Institute, 700 Ellicott Street, Buffalo, New York 14203, USA; 9 Laboratory of Molecular Biophysics, Department of Cell and Molecular Biology, Uppsala University, Husargatan 3 (Box 596), SE-75124 Uppsala, Sweden; 10 NERSC, Lawrence Berkeley National Laboratory, Berkeley, California 94720, USA; 11 Dept. de Ingeniería Aeroespacial y Mecánica de Fluidos, ETSI, Universidad de Sevilla, 41092 Sevilla, Spain; 12 Intelligent Biointegrative Systems Group, Institute of Biomaterials and Biomolecular Systems, University of Stuttgart, D-70569 Stuttgart, Germany; 13 ARC Centre of Excellence in Advanced Molecular Imaging, La Trobe Institute for Molecular Sciences, La Trobe University, Victoria 3086, Australia; 14 Physics Department, University of Wisconsin-Milwaukee, 3135 N. Maryland Ave, Milwaukee, Wisconsin 53211, USA; 15 School of Molecular Sciences and Biodesign Center for Applied Structural Discovery, Arizona State University, Tempe, Arizona 85287-1604, USA; 16 Department of Chemistry and Physics, La Trobe Institute for Molecular Science, La Trobe University, Melbourne, Victoria 3086, Australia

## Abstract

The new European X-ray Free-Electron Laser (European XFEL) is the first X-ray free-electron laser capable of delivering intense X-ray pulses with a megahertz interpulse spacing in a wavelength range suitable for atomic resolution structure determination. An outstanding but crucial question is whether the use of a pulse repetition rate nearly four orders of magnitude higher than previously possible results in unwanted structural changes due to either radiation damage or systematic effects on data quality. Here, separate structures from the first and subsequent pulses in the European XFEL pulse train were determined, showing that there is essentially no difference between structures determined from different pulses under currently available operating conditions at the European XFEL.

## INTRODUCTION

The development of serial femtosecond crystallography (SFX) using intense femtosecond-duration pulses from X-ray free-electron lasers has opened up new avenues for the measurement of macromolecular structures and macromolecular dynamics. Particular applications to date have been room-temperature measurements using micrometer-sized and smaller protein crystals, damage free determination of radiation sensitive structures, and time-resolved studies of biomolecular dynamics at physiologically relevant temperatures.^1–8^ The recently opened European X-ray Free-Electron Laser (European XFEL) free electron laser is the first facility capable of delivering millijoule X-ray pulses of femtosecond duration at a megahertz pulse repetition rate in the wavelength range useful for atomic resolution structure determination.^9^ Previous facilities were limited to a pulse repetition rate of 120 Hz by available accelerator technology. The megahertz pulse repetition rate of the European XFEL is particularly attractive because it enables the efficient measurement of the data volumes necessary for high resolution time-resolved structural studies in a comparatively short amount of data collection time. Measuring up to 3520 frames/second using currently available detector technology could enable the measurement of SFX data at almost 30 times the rate that has been possible to date, provided all pulses at megahertz pulse rates can be exploited for reliable structure determination.

**FIG. 1.**
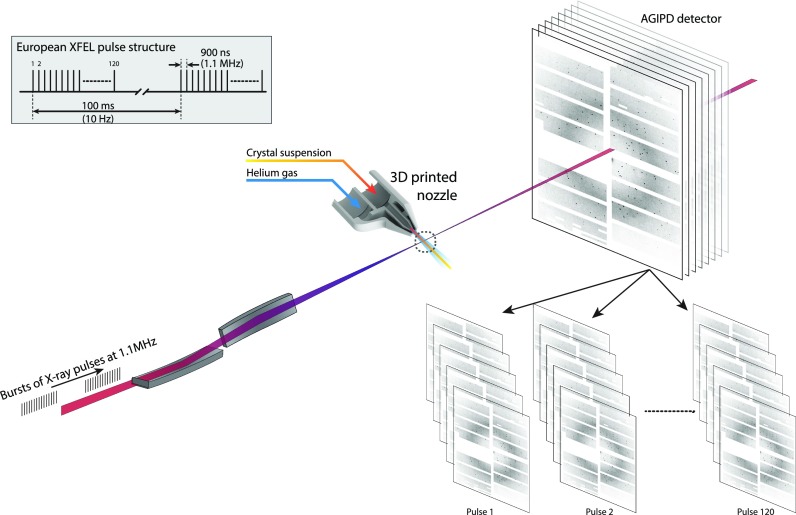
Serial crystallography to obtain a structure on each pulse of the EuXFEL pulse train.

The challenge in exploiting rapid data acquisition at the European XFEL lies in exploiting the 1.1 MHz repetition rate within a pulse train. The European XFEL does not deliver a uniform stream of X-ray pulses, rather it delivers 10 bursts (trains) of X-ray pulses/second with each burst containing up to 2700 X-ray pulses at an interpulse repetition rate of up to 4.5 MHz (1.1 MHz in this experiment), Fig. 1. The megahertz intrabunch repetition rate of the European XFEL poses unique challenges for SFX sample delivery and time resolved studies because the fresh sample must be delivered to the interaction point before the arrival of the next X-ray pulse and should not be pre-exposed or damaged on its way to the peak intensity at the focus. For time resolved studies in particular, it is important to know that delivering pulses separated by only 900 ns, nearly four orders of magnitude less time between pulses than the 8.3 ms pulse separation in experiments to date using 120 Hz pulse repetition rates, does not lead to a degradation in data quality or induce artificial structural changes. It is well known that, for example, the high dose deposited in a single focused XFEL pulse causes rapid local heating of the sample and surrounding matter to hundreds of thousands of degrees.^13^ This in turn can cause the jet to explode sending a pressure wave back up the liquid stream, creating a void that must be replenished in order to transport a fresh volume of liquid with crystals to the beam focus by the time the next pulse arrives. Photoionization in the interaction region creates high-energy electrons near the interaction region, which might be ejected from the jet. Modeling shows such electrons likely return to the (now-charged) jet column and then create a cascade of charges through collisions with atoms in the fluid.^10–12^ Solvated electrons may further diffuse through the liquid, causing changes to the oncoming sample through radiolysis. The jet may be further disrupted after the X-ray pulse has passed. X-ray induced explosion of the jet was dramatically visualized in experiments performed by Stan *et al.* at the Linac Coherent Light Source (LCLS), which imaged the effect of LCLS X-ray illumination on relatively slow and thick liquid jets of ∼20 *μ*m diameter.^13^ This heating and pressure wave,^14^ and energetic products from the ionization and vaporization, may influence the structure of protein crystals still upstream of the interaction region. The effects of any pressure wave are potentially more severe at the European XFEL due to the short time duration before the arrival of the next pulse, resulting in a smaller spacing between damaged and fresh samples at current sample delivery speeds of up to 100 m/s. Therefore, it is difficult to extrapolate jet damage studies from previous experiments performed at LCLS at 120 Hz rates.

Here, we address the question of whether the use of megahertz repetition rate X-ray pulses results in any discernible or systematic change in the solved macromolecule structure by determining independent structures for data accumulated from each pulse number in the European XFEL pulse train for lysozyme. The first SFX early experiments using 1.1 MHz pulse trains merged data from all pulses in the pulse train to determine a structure.^15,16^ Here, we concentrate on whether there are any discernible differences in structures or data quality from individual pulses in the train to determine whether the high repetition rate yields any observed structural differences or degradation in data quality. A similar experimental arrangement to that in Wiedorn *et al.*^15^ was used, however, with 120 X-ray pulses per train, reflective optics providing higher power density due to both a smaller focal spot and higher optical throughput at an average XFEL pulse energy of 1 mJ per pulse. Sample delivery was performed using liquid jets with a speed of 100 m/s and a diameter of 3 *μ*m as had previously been reported.^15^ The use of 120 X-ray pulses per train and a pulse train repetition rate of 10 Hz for this experiment results in a data acquisition rate of 1200 frames/second, 10 times higher than previously available at the LCLS. Over 1.4 × 10^6^crystal lattices were measured from the well-known reference test system lysozyme, enabling us to divide the data into separate datasets for each pulse number, Fig. 1, and to determine separate structures for each pulse number in the pulse train. At current experimental conditions, no significant or systematically interpretable difference in data or reconstructed structures for each pulse in the pulse train was found. Since the structures from each pulse are identical, it is possible to merge data from all 120 pulses together, at which point we demonstrate the collection of sufficient data for structure determination in approximately one minute using the currently available 1200 pulses/second.

## MATERIALS AND METHODS

The experiment was conducted at the SPB/SFX (Single Particles, Clusters, and Biomolecules and Serial Femtosecond Crystallography) instrument of the European XFEL^17^ in September 2018 during experiment number p002120 using a similar instrument configuration as described for previous experiments.^15^ The main difference in the instrument configuration was the use of reflective optics in Kirkpatrick-Baez like geometry for X-ray focusing instead of compound refractive lenses, resulting in a higher beamline transmission and a smaller focal spot,^18^ Fig. 1. X-ray pulses with a mean photon energy of 9.35 keV (1.33 Å wavelength), a mean pulse energy of 1.04 mJ, and pulse length of approximately 15 *μ*m (50 fs duration derived from the electron bunch length) were focused by reflective Kirkpatrick-Baez optics in a 4-bounce configuration located 20 m upstream of the interaction region^18^ into a focal spot of 2 × 3 *μ*m^2^ full width at half maximum (FWHM) at the SPB/SFX interaction region. The European XFEL pulse structure for this experiment comprised 120 X-ray pulses at a 1.1 MHz repetition rate repeating at 10 Hz for a total of 1200 pulses/second. This pulse structure provides a factor of 10 increase in measured pulses/second than available at other currently operational hard X-ray XFELs. Diffraction from each X-ray pulse was measured using a 1 megapixel Adaptive Gain Integrating Pixel Detector (AGIPD)^19^ located 118 mm downstream of the interaction region giving 1.9 Å resolution at the edge of the detector.

Microcrystals of hen egg white lysozyme (HEWL) of less than 5 × 5 × 5 *μ*m^3^ size were introduced into the X-ray interaction region in a liquid jet of 2–3 *μ*m diameter at speeds of 100 m/s produced by a flow focusing nozzle under similar operating conditions as described in Wiedorn *et al.*^15,20^ We had previously established that jets of this speed recover in time for the next X-ray pulse at megahertz repetition rates^15^ and aimed for similar operating conditions in this experiment. Data were collected at room temperature, and the average dose for each crystal was estimated to be 14 MGy using RADDOSE-3D version 2.1.^21^ The aim was to collect enough data to obtain a structure of lysozyme from each pulse in the train.

## RESULTS AND DISCUSSION

**FIG. 2.**
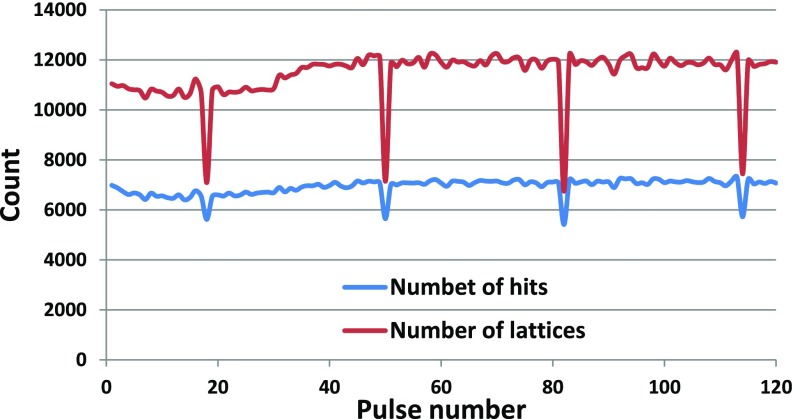
Number of hits as a function of pulse ID across all data. The hit fraction as a function of pulse number indicates that crystals are hit randomly on any pulse within the megahertz European XFEL pulse train and not only on the first pulse in the pulse train. The decreased hit fraction for pulse numbers 18, 50, 82, and 114 appears to arise from a systematic detector artifact in certain AGIPD memory cells for a portion of the data.

A total number of 16 654 608 data frames were collected from HEWL crystal solution at 1200 pulses/second and a peak pulse repetition rate of 1.1 MHz, of which 830 514 images (5%) were found to contain diffraction from a crystal as identified by Cheetah,^22^ which was also used to apply the AGIPD calibration. Frames containing crystal diffraction patterns were distributed roughly evenly throughout the pulse train, Fig. 2. This is consistent with the results from previous experiments using 15 pulses per train and liquid jet speeds of 100 m/s^15^, demonstrating the same trend for 120 pulses per train. We note the decreased hit fraction for pulse numbers 18, 50, 82, and 114, which arise from a systematic detector artifact in these AGIPD memory cells. Some deviation from the expected straight line is explained in Methods data analysis section.

**FIG. 3.**
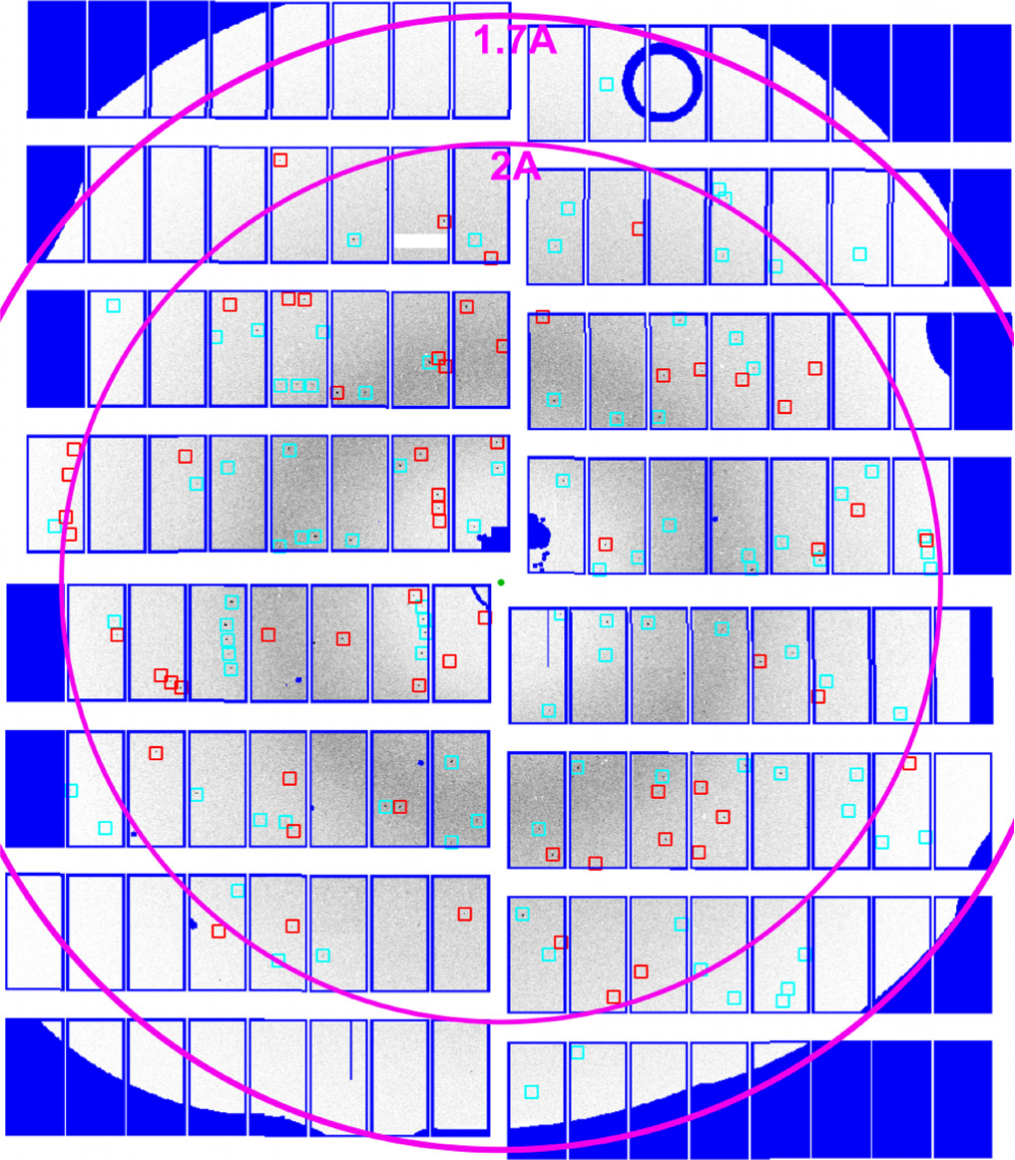
Multiple lattices measured in a single pulse. A single indexed diffraction pattern from HEWL shows multiple lattices indeed by XGANDALF, and red and blue squares represent predicted Bragg peaks for two crystal lattices. On average 1.6 lattices per frame containing crystal diffraction pattern was determined. Dark blue is the masked region of the detector.

From the identified hits, 91% (782 500) could be indexed using the CrystFEL software suite,^23,24^ yielding 1 374 785 indexed crystal lattices for structure determination when allowing multiple lattices per image (“–multi” option in CrystFEL). The presence of indexed multiple lattices in some diffraction patterns was verified by inspection of predicted peak locations for multiple lattices in frames identified by CrystFEL as containing multiple lattices. For example, Fig. 3 shows a sample diffraction pattern containing multiple lattices where spot prediction matches observed Bragg peak locations. The presence of multiple hits could be either due to random clustering of crystals in suspension or due to crystals adhering together, either of which could produce multiple lattices in a single frame consistent with the indexing results.

Merging all reflection intensities using the program “partialator” from CrystFEL in point group 4/mmm produced a dataset with an error metric R_split_ of 1.8 and CC^*^ of 0.9999 to 1.6 Å resolution. Diffraction at high resolution was limited by the exit aperture of the differential pumping catcher (see dark blue mask in Fig. 3), suggesting that a higher resolution could be achieved in the future using either shorter wavelength X-rays or adjustment of the SPB/SFX sample delivery hardware to accommodate a larger clear solid angle toward the downstream detector.

**FIG. 4.**
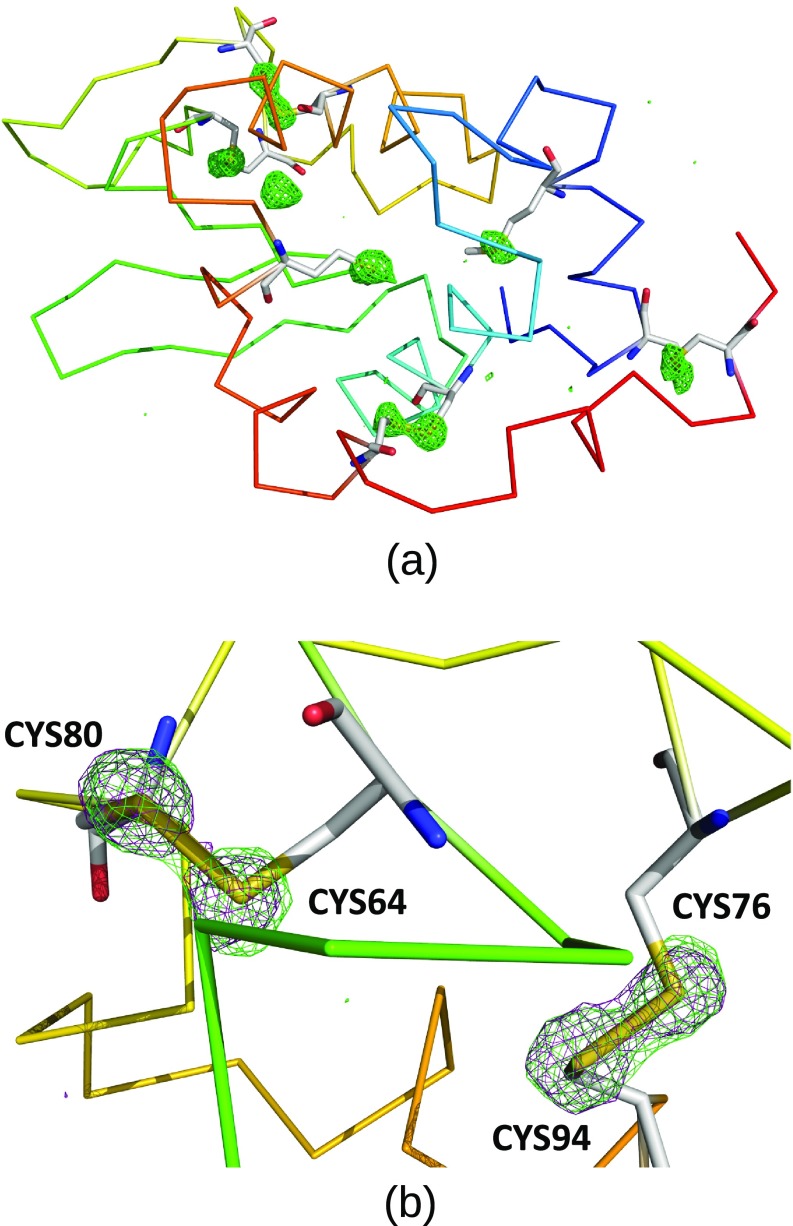
Phased anomalous Fourier difference map contoured at 3.5 sigma at 9.3 keV using all data to 1.6 Å resolution. (a) Whole protein and (b) zoom in on selected residues. Green mesh is the anomalous difference map for whole data and magenta—for a half the dataset. Extra green density in (a) is probably Cl^−^ ions. Residues in (b): CYS80-CYS64 and CYS94-CYS76.

The structure from each pulse was determined by molecular replacement using Phaser within “Phenix”^25^ software suite and structural model of lysozyme from Wiedorn *et al.*^15^ (PDB^26^ accession code 6FTR). Refinement with phenix.refine yields in a structural model with R_work_/R_free_ of 14.2/16.0 to 1.6 Å resolution, Table I. Merging data in a noncentrosymmetric point group shows sufficient anomalous signals to enable *ab initio* automatic structure determination by native Sulfur SAD phasing (CFOM of 39.1 in SHELXD^27^ using a 1.9 A resolution cutoff). Automatic model building and refinement using the CRANK2-pipeline in CCP4^28^ software suite followed by automatic model building and refinement using AutoBuild^29^ in Phenix yielded a structural model with R_work_/R_free_ of 15.4/17.3 using a resolution cutoff in a final autobuilding of 1.6 Å. The anomalous difference density map is shown in Fig. 4

**TABLE I.** SFX data and refinement statistics for combined data.

Parameter	Lysozyme (all)	
Merged into point group	4/mmm	422
Photon energy (mean value) (eV)	9300	
X-ray focus, FWHM (*μ*m)	2 × 3	
Pulse energy at sample (70% beamline transmission) (*μ*J)	800	
Pulse length (fs)	<40	
Space group	P 4_3_ 2_1_ 2	
Unit cell		
a, b, c (Å)	79.6, 79.6, 38.3	
α, β, γ (°)	90, 90, 90	
Number of hits/indexed lattices	782 500/1 374 785	
Number of unique reflections	16 454	30 336
Resolution range (Å)	15.66 −1.6 (1.68 −1.6)	15.37 − 1.6 (1.68 −1.6)
Completeness (%)	99.9 (99.7)	99.8 (98.7)
Multiplicity/redundancy	9827.1 (64.9)	5327.5 (37.3)
R_split_ (%)	1.80 (39.11)	2.35 (45.55)
I/σ(I)	41.5 (4.0)	30.7 (3.1)
CC_1/2_	0.99970 (0.82438)	0.99953 (0.76752)
CC^*^	0.99992 (0.95065)	0.99988 (0.93192)
CC_ano_	…	0.24617 (0.096)
Wilson B-factor (Å^2^)	19.48	19.45
R_work_	0.142 (0.239)	0.154 (0.284)
R_free_	0.160 (0.258)	0.175 (0.292)
RMSD bonds (Å )/RMSD angles (deg)	0.009 / 1.30	0.010 / 1.53
Ramachandran favored	99.21	99.21
Ramachandran allowed	0.79	0.79
Ramachandran outliers	0	0
Average B-factor	25.44	25.83
Macromolecules	24.16	24.88
Ligands	49.94	…
Solvent	34.47	37.63
CXIDB data deposition	CXIDB ID-98	

**FIG. 5.**
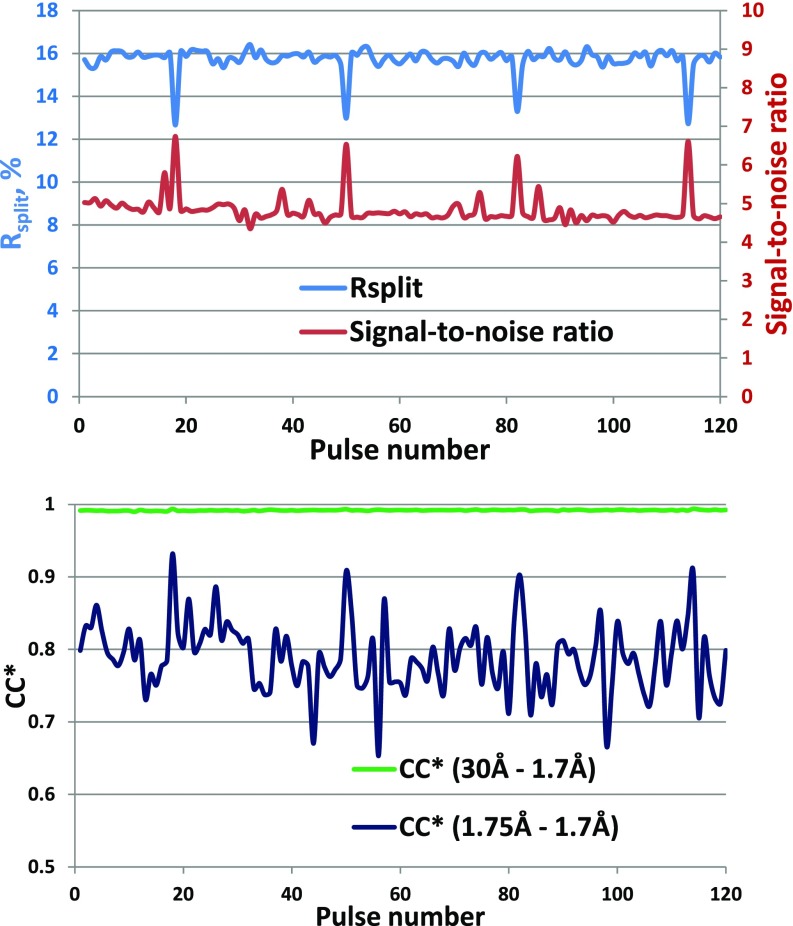
Data quality metrics R_split_ and signal-to-noise ratio (left) and CC^*^ (right) as a function of pulse number in the train.

**FIG. 6.**
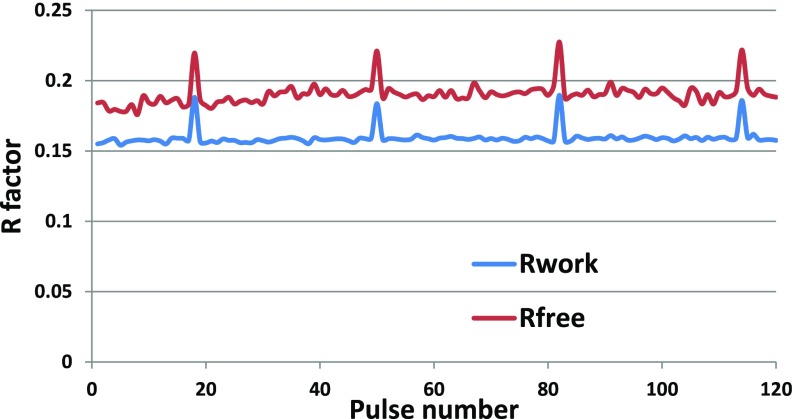
R_free_ and R_work_ as a function of pulse number in the train.

**FIG. 7.**
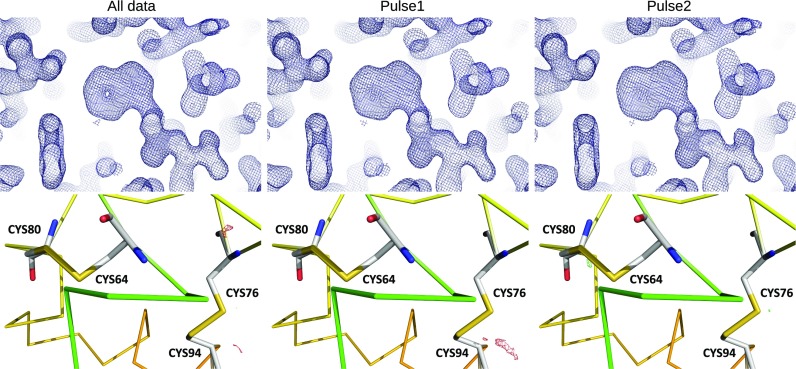
Comparison of the structure from all data to structures from the first and second pulses in the pulse train. The 2Fo-Fc map contoured at 1.5 sigma (top row) and part of the structure with Fo-Fc difference maps contoured at 3 sigma (bottom row) determined from data merged from all pulses (first column) and maps determined from data only for the first and second pulses in the pulse train (second and third columns, respectively). Structures for all pulses in the pulse train are included in the data deposition.

The data were then divided into separate datasets for each pulse in the pulse train to assess whether there are any systematic pulse train effects on structure quality. Merging data from each pulse separately, the data quality metrics R_split_, CC^*^, and I/sigma(I) show no meaningful degradation in data quality within the pulse train, Fig. 5. With almost 1.4M indexed lattices approximately evenly distributed through the pulses in the train, we obtain indexed lattices between 11 000 and 12 000 for each pulse, sufficient to obtain a structure for each pulse. The high resolution cutoff for each of the 120 datasets was set to 1.7 Å—at this resolution, all datasets are still reliable (see CC^*^ for the highest shell in Fig. 5). Table 3 in the Supplemental Material section shows the results of applying the same merging and refinement procedure described above to data from each individual pulse in the pulse train. The data in Table 3 show that refinement statistics are consistent for all pulses in the pulse train. Figure 6 plots the R_work_ and R_free_ metrics as a function of pulse ID, which shows that the values are very consistent through the pulse train except for pulses 18, 50, 82, and 114. Excluding these pulses that contain artifacts from bad memory cells from the all-pulse dataset had no discernible impact on the structure nor were the all-pulse quality metrics degraded. Inspection of structures across pulses shows no statistically significant differences, even between the first and subsequent pulses (see the Methods structure determination section). For example, Fig. 7 shows structure and difference maps determined from the first and second pulses in the pulse train compared to the structure determined from merging all pulses together.

During one 30 min period of data collection, we were able to collect over 2 million detector frames of which 190 000 images contained crystal diffraction patterns, yielding 332 482 indexed crystal lattices (runs 96–98, one run is 10 min of data collection). This raises the prospect of elucidating small changes in structure factors by the accumulation of statistics in orders of magnitude less time than previously possible, for example, in time resolved experiments for producing molecular movies, for the determination of structures from weakly diffracting crystals^7^ in which averaging of large datasets is required to obtain a sufficient signal-to-noise ratio (SNR) for structure determination. For example, integrating data across all 120 pulses in the pulse train, a SNR of 15 was obtained from a 10 min data run containing 117 415 indexable lattices. This increased to a SNR of 25 for a 30 min run containing 332 482 lattices and a SNR of 48 for the whole dataset consisting of 1 374 785 indexable lattices. Data metrics for different point groups as well as different resolution cutoffs and smaller portions of the data are summarized in Table II.

**TABLE II.** SFX data and refinement statistics for all pulses merged together.

	Data collection		Data quality			Refinement					
Dataset	Number of hits	Number of lattces	CC^*^	R_split_ (%)	SNR	R_work_	R_free_	RMSD bonds (Å)	RMSD angles (deg)	B-factor (Å^2^)	N water
Run 97 only (approximately 10 min)	67 090	117 415	0.9993	4.84	15.17	0.140	0.164	0.009	0.99	25.3	67
Runs 96–98 (approximately 30 min)	189 960	332 482	0.9998	2.86	25.59	0.137	0.159	0.009	0.97	25.8	64
All data to 1.7 Å	830 514	1 374 785	0.9999	1.51	48.74	0.135	0.157	0.009	0.98	25.9	70
All data to 1.6 Å	830 514	1 374 785	0.9999	1.80	41.46	0.142	0.160	0.009	1.00	25.4	64
All data to 1.6 Å merged in PG 422	830 514	1 374 785	0.9999	1.99	36.18	0.153	0.171	0.006	0.89	25.5	81

There is no strict requirement on the number of indexed patters needed for successful structure determination using molecular replacement. For example, the beta-lactamase structure previously determined at European XFEL^15^ was solved using 14 000 indexed lattices. Under current operating conditions, sufficient data could be collected in less than two minutes at an ∼10% hit fraction using less than 60 *μ*l of crystal solution enabling substrate screening using mix-and-inject SFX as previously noted.^15^ Naturally, data quality improves through improved statistics as more data are measured. For example, using only 10 min of data collection (run 97), R_work_/R_free_ of 0.1399/0.1640 were obtained from 117 415 crystals in 67 090 diffraction patterns, compared to R_work_/R_free_ of 0.1368/0.1587 from 332 482 crystals in 189 960 diffraction patterns from 30 min of data collection and R_work_/R_free_ of 0.1347/0.1570 from 1 374 785 crystals in 830 514 diffraction patterns from the whole dataset to 1.7 Å resolution, Table II.

## CONCLUSIONS

Structures determined from each pulse number in the European XFEL pulse train result in essentially identical data quality and refinement metrics for lysozyme microcrystals under the exposure and sample delivery conditions available in this experiment. Conclusions one can draw from this result are as follows: (1) The structures from each pulse in the pulse train show no significant differences from each other to 1.7 Å resolution; thus, it is possible to measure time-resolved structures in the future using each pulse in the pulse train without having to account for changes due to the megahertz repetition rate under the exposure conditions available in this experiment; (2) since the structures determined from each pulse are essentially identical, it is possible to merge data from all pulses in the pulse train into one dataset without having to account for changes due to the megahertz repetition rate; and (3) it is now more feasible than ever to improve data quality by averaging of large datasets for time resolved experiments or using small or weakly diffracting crystals, obtaining sufficient SNR for structure determination in reasonable experiment time;^7^ alternatively (4) sufficient data for a molecular replacement structure determination using 1200 pulses/second can enable faster completion of datasets for structure determination by SFX as previously predicted.^15^ Observation that no significant changes through the pulse train were observed is valid only for current experimental conditions, including reflective optics for a micrometer scale focal spot and HEWL as a test sample. One should still be careful to further verify these conclusions when performing experiments at higher peak pulse powers, using highly absorbing metal centers, or with slower liquid jet delivery than used here. Nevertheless, the results presented here form a useful baseline demonstrating negligible pulse train damage for reference in future experiments and demonstrate the potential for European XFEL to measure data using X-ray pulses with a 1.1 MHz repetition rate for the collection of datasets with higher statistics than previously possible in the same measurement time.

## Figures and Tables

**FIG. 8.**
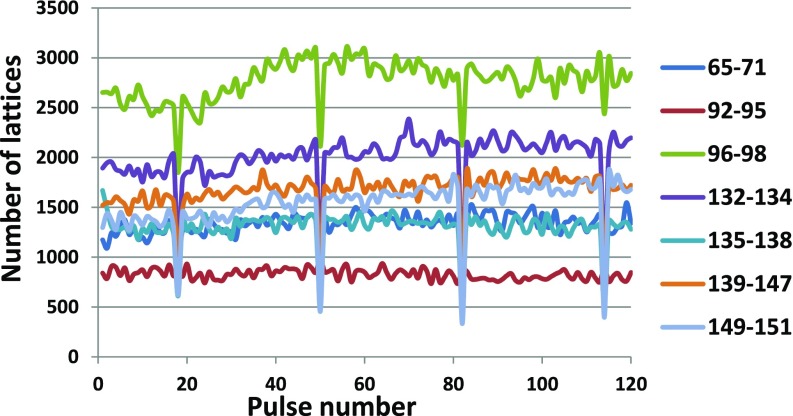
Number of lattices per pulse for different blocks of runs. Small deviation from linear behavior for some plots might be due to different intensities for each pulse in the train. This intensity distribution was changing during the 5 days of the experiment.

**FIG. 9.**
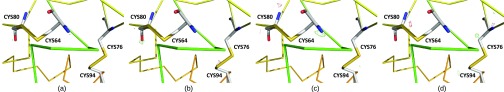
Isomorphous Fo-Fo difference maps contoured at 3 sigma, comparing observed data from the first pulses only with observed data from (a) 2nd pulses, (b) 3rd pulses, (c) 60th pulses, and (d) 120th pulses.

**FIG. 10.**
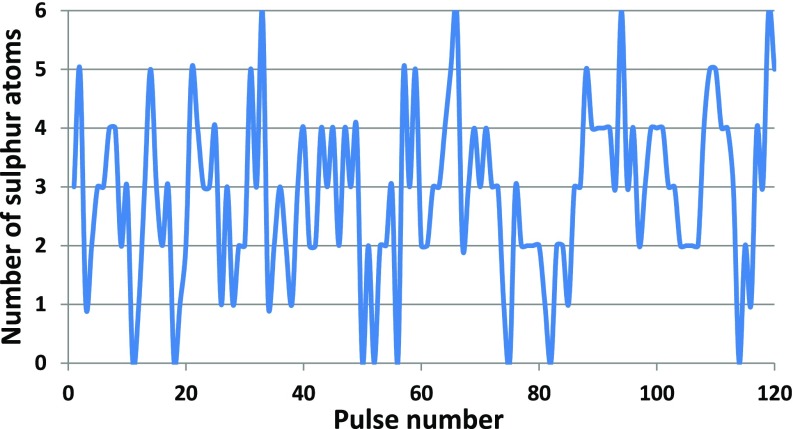
Number of sulfur anomalous difference map peaks per pulse. Only peaks with a height greater than 3 sigma were considered.

## APPENDIX: METHODS

### Sample preparation

Samples were prepared in the same manner as previously reported.^15^ Briefly, crystals of hen egg-white lysozyme (HEWL) were grown by the rapid-mixing batch method.^30^ Tetragonal crystals with a size of each side 2–5 *μ*m were obtained by adding three parts of precipitant [1.2 M NaCl, 36%(*v*/*v*) ethylene glycol, 15%(*w*/*v*) PEG 4000, 50 mM sodium acetate buffer pH 3.5 filtered through a 450 nm filter] to one part of HEWL (Sigma–Aldrich; dissolved to 126 mg ml^−1^ in 50 mM sodium acetate buffer pH 3.5 and filtered through a 100 nm filter) at 1 °C (ThermoStat C, Eppendorf, Germany). The resulting mixture was immediately subjected to rapid mixing and incubated for 30 min at 1 °C.^31^ Crystal sizes were determined by image analysis by optical microscopy. Crystals were resuspended before injection to yield a homogenous suspension of HEWL microcrystals.

### Instrumentation

Experiments were performed at the SPB/SFX serial femtosecond crystallography instrument^32^ at the European XFEL X-ray free electron laser in September 2018 as a part of proposal p002120 using a similar configuration as used in Wiedorn *et al.*^15^ as described in the main text. The size of the focal spot in the interaction region was estimated to be 2 × 3 *μ*m^2^ FWHM based on optical imaging of single shots using a 20 *μ*m thick Ce:YAG screen. Diffraction from the sample was measured using an AGIPD 1M located 117.7–118.6 mm downstream of the sample interaction region, with the unused direct beam passing through a central hole in the detector to a beam stop further downstream.

HEWL microcrystals were delivered to the X-ray interaction region in a similar manner as used in Wiedorn *et al.*^15^ using a 3D-printed gasdynamic virtual nozzle^33–35^ in which a liquid stream is focused and accelerated by the virtual orifice created by a copropagating helium gas flow or a 3D-printed double flow focusing nozzle.^35^ The speed of the liquid jet was estimated to be 100 m/s based on measured flow rates during the experiments and offline speed measurements under the same operating conditions using similar printed 3D nozzles.^20^ The liquid jets were positioned in the interaction region by mounting nozzles on a moveable “nozzle rod,” which held the jets just above the X-ray focal position and aligned to the X-ray beam using an in-line microscope viewing system.

### Data analysis

Experimental progress was monitored online using OnDA^36^ for serial crystallography. Of the 16 654 608 diffraction patterns collected during HEWL data acquisition runs used for final analysis, 830 514 images (5%) were determined by Cheetah^22^ to contain crystal diffraction patterns (peakfinder8, minSNR = 8, minADC = 50, minPix = 1, minPeaks = 20) using the calibration process described in Wiedorn *et al.*^15^ Careful masking of shadowed and unreliable regions of the detector was performed on run-by-run basis. Independent masks were used for peakfinding to avoid false peaks, for example, due to ice formation. Indexing was performed using CrystFEL version 0.8.0, using the indexing package XGANDALF^37^ on peaks found by Cheetah.^22^ Indexing of multiple lattices per image resulted in a higher number of indexed lattices than the number of input images. Experimental geometry, especially the detector distance, was refined for 8 blocks of runs (with consistent parameters) using the program geoptimiser.^38^ Merging and scaling of the Bragg peaks intensities were performed using the partialator program from CrystFEL. To ensure similar processing for each of the 120 pulses, the option custom-split was used. To avoid the integration of noise for weakly scattering patterns, reflections were included up to 0.6 nm^−1^ above a conservative resolution estimate for each crystal (–push-res = 0.2). MTZ-files for crystallographic data-processing were generated from CrystFEL merged reflection data files using f2mtz of CCP4.^28^ Figures of merit were calculated using compare_hkl (Rsplit, CC_1/2_, CC^*^) and check_hkl (SNR, multiplicity, completeness), both a part of CrystFEL. The total number of indexed crystals 1 374 785 resulted in 11 000–12 000 crystals for each of the 120 pulses. The change in the number of measured “hits” per pulse (Fig. 2 and Table III in supplemental material) might be explained by the change of the intensity of x-ray pulses within each train. The distribution of the pulse intensity inside train was changing during the whole experiment, which was observed at the monitors in the control hutch. This is reflected in the change of number of “hits” for different runs measured during the 5 days beamtime—Fig. 8(a). Unfortunately, information about per pulse intensity (or more precisely electron bunch energy) was not saved during the experiment at that time. Reconstruction of the equivalent number of patterns per pulse (6500) was also performed but showed no noticeable difference to the results obtained from all data.

For RADDOSE-3D calculations, the following parameters are used: cubic crystal of 5 × 5 × 5 *μ*m^3^ with 10 pixels/*μ*m, providing PDB code 4ET8, Gaussian beam with 4.8 × 10^24^ photons/s in 2 × 3 *μ*m^2^ FWHM size at 9.3 keV and 1 × 10^13^ s exposure.

### Structure determination

A structural model of lysozyme from Wiedorn *et al.*^15^ (PDB accession code 6FTR) was used as a starting model for molecular replacement in Phaser.^39^ Phaser was used to orient the model according to the orientation of the data. Using the structural model directly in phenix.refine (for which the isomorphicity of the crystals would have allowed) failed. For all subsequent refinement, the same reoriented and rerefined PDB-model based on 6FTR with all solvent molecules was used, alternative conformations and ligands as input for refinement. A simple bash script generated the same R_free_-flags for all datasets (copied from 6FTR and extended to full resolution) and the same refinement parameters used for the overall dataset were used for all single-pulse datasets, as well as the same starting model. Note that for this publication, we did not strive to find the best refined model, and it was for us more important to have comparable results. Hence, the structural model was not optimized in manual rebuilding cycles combined with automated maximum-likelihood refinement. The same input-model, same R_free_-flags, and same refinement parameters in Phenix were used. Isomorphous Fo-Fo difference maps for the structures reconstructed from the first and several subsequent pulses were calculated using the Isomorphous difference map tool in Phenix and are presented in Fig. 9.

For anomalous sulfur SAD-phasing for the combined dataset, the reflection list using point group 422 in partialator was generated. Point group 422 was used to avoid merging of Friedel pairs of reflection in point group 4/mmm. MTZ files were generated as described above and phenix.french_wilson was used to generate MTZ-files with F+/F− columns. Anomalous phasing was attempted using the CRANK2-pipeline in CCP4 employing the SHLEXC/D/E pipeline and the programs Parrot, Buccaneer,^40^ and REFMAC5.^41^ For the successful phasing case, the MTZ-file and initial model from CRANK2 were exported to AutoBuild in Phenix for advanced automatic model building. Data metrics for 4/mmm and 422 point groups are summarized in Table II. Figure 10 also demonstrates the number of sulfur anomalous difference map peaks above 3 sigma per pulse. It shows no clear trend depending on the pulse number, which also suggests no apparent damage for different pulses in the train. The phased anomalous Fourier difference map was calculated with phenix.find_peaks_holes from Phenix using a custom script.

### Data deposition

Data have been deposited with the Coherent X-ray Imaging Data Bank^42^ with deposition ID 98. These include the following:

• Crystal hits used for indexing
• Stream files for all data and for data separated into each pulse.
• MTZ and PDB files for all data and separated by pulse ID

We do not see a point of depositing 120 PDBs to the Protein Databank, and so we have deposited all resulting data for all 120 pulses to the CXIDB only.

### SUPPLEMENTARY MATERIAL

See supplementary material for SFX data and refinement statistics separated by pulse ID in the pulse train.
